# Adsorption Property and Morphology Evolution of C Deposited on HCP Co Nanoparticles

**DOI:** 10.3390/molecules29194760

**Published:** 2024-10-08

**Authors:** Lili Liu, Yujia Shi, Jiamin Rong, Qiang Wang, Min Zhong

**Affiliations:** 1 School of Semiconductor and Physics, North University of China, Taiyuan 030051, China; 13417536993@163.com (Y.S.); rongjiamin@126.com (J.R.); 2 National Key Laboratory of High Efficiency and Low Carbon Utilization of Coal, Institute of Coal Chemistry, Chinese Academy of Sciences, Taiyuan 030001, China; wqiang@sxicc.ac.cn; 3 Chemical Synthesis and Pollution Control Key Laboratory of Sichuan Province, College of Chemistry and Chemical Engineering, China West Normal University, Nanchong 637002, China; m_825179076@sina.com

**Keywords:** density functional theory, morphology evolution, Co nanoparticles, deposited carbon

## Abstract

Despite extensive studies of deposited carbon in Fischer–Tropsch synthesis (FTS), an atomic-level comprehension of the effect of carbon on the morphology of cobalt-based FTS catalysts remains elusive. The adsorption configurations of carbon atoms on different crystal facets of hexagonal close-packed (hcp) Co nanoparticles were studied using density functional theory (DFT) calculations to explore the interaction mechanism between C and Co surfaces. The weaker adsorption strength of C atoms on Co(0001), Co(10-10), and Co(11-20) surfaces accounted for lower diffusion energy, leading to the facile formation of C dimers. Electronic property analysis shows that more electrons are transferred from Co surfaces to C atoms on corrugated facets than on flat facets. The deposition of carbon atoms on Co nanoparticles affects surface energy by forming strong Co-C bonds, which causes the system to reach a more energetically favorable morphology with an increased proportion of exposed Co(10-12) and Co(11-20) areas as the carbon content increases slightly. This transformation in morphology implies that C deposition plays a crucial role in determining the facet proportion and stability of exposed Co surfaces, contributing to the optimization of cobalt-based catalysts with improved performance.

## 1. Introduction

Cobalt-based catalysts [1,2] have been characterized by higher activity, higher selectivity for long hydrocarbon chains, and lower water–gas shift activity, making them the ideal catalysts for Fischer–Tropsch synthesis (FTS) reactions [3,4]. The surface structure and morphology of Co-based catalysts, as well as their catalytic activity, can be significantly affected by pretreatment conditions, reaction environments, and reaction intermediates [5,6]. The intermediates typically contain deposited carbon [7,8], which remains a significant challenge that affects the stability and longevity of Co-based catalysts.

Deposited carbon generally exists in two forms in metallic Co: one is located on the Co surfaces in the form of carbon monomers [9], and the other permeates into the crystal lattice, reacting with cobalt to form carbides [10,11], such as Co_2_C and Co_3_C. The permeation of carbon occurs in hydrogen-deficient syngas and at low temperatures, inducing distortion in the cobalt lattice and exhibiting a relatively inert kinetic behavior [12,13]. In contrast, the formation of surface carbon is easier and more abundant under reaction conditions, which has captured the growing interest of researchers.

Many studies suggested that the presence of carbon on Co surfaces is widely recognized as a critical element that impacts the performance of cobalt-based catalysts [14,15]. It was reported that carbon deposition on Co surfaces blocked the active sites of a catalyst that were responsible for deactivation [16,17,18]. Meanwhile, graphitic carbon was observed to limit the diffusion of longer-chain products. The optimization of the catalyst structure through decarburization at specific temperatures has been demonstrated to enhance CO conversion rates and adjust the selectivity of the produced hydrocarbon [19]. Alternatively, the aggregation of deposited carbon, assumed to form a protective film that inhibits the oxidation and corrosion of Co surfaces effectively was suggested to enhance the stability and prolong the lifetime of Co-based catalysts [20]. The graphitic carbon shell in Co@C core–shell nanoparticles prepared by a low-temperature carbonization process was shown to restrict the aggregation of cobalt nanoparticles, thereby enhancing catalytic performance with a C_5+_ selectivity of up to 56.8%. Lu et al. (2023) elucidated the impact of various carbon deposits on cobalt catalysts in Fischer–Tropsch synthesis, identifying that atomic carbon exhibits high CO conversion, low methane selectivity, and excellent stability, while polymeric and graphitic carbons are detrimental for FTS [21]. Despite the growing number of concerns about the effect of deposited carbon on the catalytic activity of Co-based catalysts [22,23], there are still few regarding the interaction mechanisms between carbon and Co-based catalysts, as well as the influence of carbon on the morphology of cobalt-based catalysts given that the performance of Co-based catalysts is highly dependent on their structure, which in turn unveils their underlying nature [24,25,26]. Therefore, exploring the morphology evolution induced by carbon on Co catalysts is crucial for understanding the role carbon plays in Co-based catalysts for FTS reactions and for the strategic optimization of their catalytic efficiency.

Advanced experimental techniques, including in situ spectroscopic analysis [27,28], transmission electron microscopy (TEM) [29], scanning tunneling microscopy (STM) [30], and high-resolution TEM (HRTEM) [31], have made significant progress in probing the intricate structure dynamics of Fischer–Tropsch synthesis (FTS) catalysts under reaction conditions. Qin et al. (2023) implemented a 5% CO pretreatment to deposit surface carbon onto single-crystal cobalt catalysts. They observed that the surface carbon, distributed in a screen-like pattern, significantly enhances the adsorption of bridge-bonded CO molecules, thereby promoting the formation of long-chain hydrocarbons [32]. Liu et al. (2024) found a significant dependence of anti-carbon-deposition stability on the crystallographic structure and morphology of Co catalysts. It suggests that hexagonal close-packed (hcp) Co with specific facets (10-10), (10-12), and (10-11) is desirable for achieving high stability and activity during the FTS process [33]. However, there remain challenges in the deliberate determination and characterization of the morphology and specific surfaces exposed on Co-based catalysts with different contents of deposited carbon formed during FTS reactions. Additionally, the elucidation of the mechanism of such deposited carbon on the catalyst and the structure–activity relationships of Co-based catalysts in FTS are still subjects for further research [16]. Theoretical calculations [34,35,36] have been employed as an important instrument that enables a more exhaustive understanding of the mechanisms at the atomic level of the interaction between deposited carbon and Co-based catalysts, and it is expected to guide the rational design of advanced Co catalysts endowed with enhanced performance and stability.

In this study, density functional theory (DFT) calculations were performed to study the interaction mechanism between C and hcp crystalline-phase Co and the morphology evolution of hcp cobalt nanoparticles induced by surface C. The adsorption configurations, electronic properties, and diffusion behavior of C atoms on six surfaces of hcp Co nanoparticles were analyzed. Based on the surface energies of C deposited on the exposed facets of hcp Co, the morphology of hcp cobalt nanoparticles on which different C contents were deposited was predicted by the Wullf construction. Our findings indicate that as the carbon content increases to 1/16, there is a notable alteration in the relative proportions of the Co(0001), Co(10-12), and Co(11-20) facets, suggesting that carbon deposition can modulate the surface energy and active sites of the catalyst by forming strong Co-C bonds, thereby impacting the catalyst’s stability and activity. Our work provides theoretical guidance for the design and optimization of cobalt-based catalysts with superior performance.

## 2. Results and Discussion

### 2.1. C Adsorption on HCP Co Surfaces

**Figure 1:**
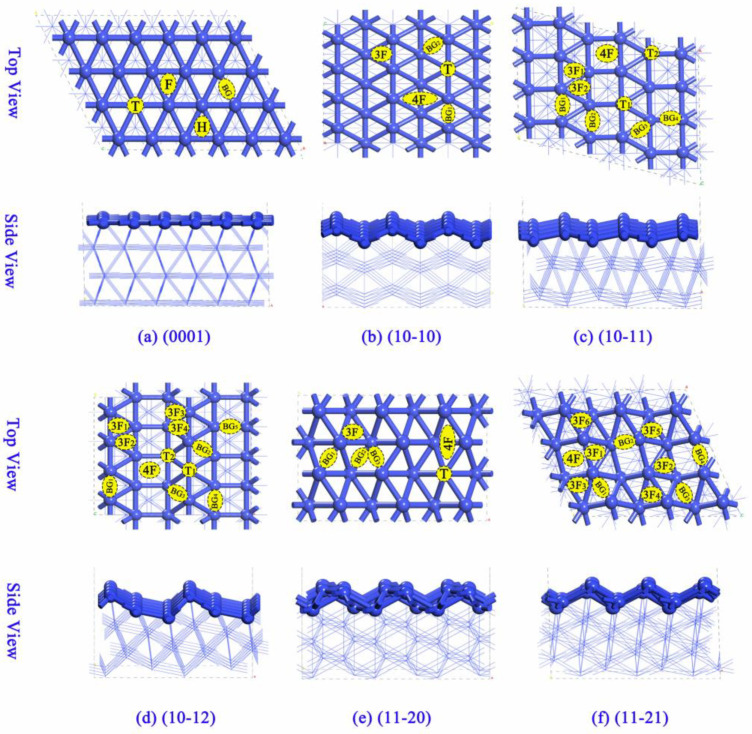
The surface structures and the corresponding adsorption sites for (**a**) Co(0001), (**b**) Co(10-10), (**c**) Co(10-11), (**d**) Co(10-12), (**e**) Co(11-20), and (**f**) Co(11-21) surfaces. The Co atoms are colored blue.

To study the adsorption properties of the C atom on hcp Co surfaces, including Co(0001), Co(10-10), Co(10-11), Co(10-12), Co(11-20), and Co(11-21), we focused on ideal hcp Co surfaces with larger exposed areas. As shown in Figure 1, all possible sites for C adsorption (T for the top site, BG for the bridge site, F for the face-centered cubic (fcc) site, H for the hcp site, and nF for the *n*-fold site) were tested to determine the most stable configurations. The F (fcc) site, H (hcp) site, and 3F site are all located at the center of a three-fold coordinated site, where the adsorbate interacts with three surface atoms at once. For the Co(0001) surface, the second-layer atoms are precisely positioned at the centers of both the first- and third-layer atoms, creating a hexagonal arrangement. This site is commonly referred to as the hexagonal close-packed hole, denoted as the H site. Distinct from the H site, the face-centered cubic hole is marked as the F site. For convenience in labeling, we use the common F and H sites on the Co(0001) surface rather than the 3F site. 

The most stable adsorption site is the H site for a single C atom on the Co(0001) surface, while the four-fold site is most stable on Co(10-10), Co(10-11), Co(10-12), Co(11-20), and Co(11-21) surfaces. The optimized adsorption energies and key parameters for C adsorption on six Co surfaces are given in Table 1. The minimum adsorption energy for a single C atom is −7.04 eV on the Co(0001) surface, while the maximum adsorption energy is −8.29 eV on the Co(10-11) surface. For other surfaces, the adsorption energy of the C atom ranges from −7.13 to −7.98 eV. These values are in good agreement with the calculated results in the literature [37]. For the wrinkled Co(10-10), Co(10-11), Co(10-12), Co(11-20), and Co(11-21) surfaces, the coordination number of C atom at the stable site is larger than that on the flat Co(0001) surface, and the C-Co average bond length is also increased. For example, the average C-Co bond length on the Co(11-20) surface with 1.98 Å is larger than that (1.78 Å) of the Co(0001) surface.

As the amount of deposited carbon increases, C atoms show distinct behaviors on different hcp Co surfaces, some aggregating while others are dispersing. Consequently, it is crucial to explore the stability of the adsorption configurations as the number of C atoms increases. The second C atom was adsorbed based on the stable adsorption site of the first C atom, and only the C_2_ structure with the lowest energy was discussed. The optimized configurations, adsorption sites, average adsorption energies, and key parameters of two C atoms on Co surfaces are listed in Table 1.

As more C atoms are deposited on Co(0001), Co(10-10), Co(11-20), and Co(11-21) surfaces, the second C atom prefers to adsorb on the meta-stable site (F or 3F site), bonding with the first deposited carbon to form the C_2_ dimer. The corresponding average adsorption energies of the C_2_ dimer are lower than those of single C atoms, suggesting that C atoms are favored to aggregate on these four Co surfaces. For the Co(10-11) and Co(10-12) surfaces, the second C atom is dispersed and adsorbed on the most stable 4F site instead of interacting with the first C atom. The corresponding average adsorption energies of the C_2_ configuration are higher than those of single C atoms, indicating that the C atoms on these two Co surfaces tend to be in a dispersed form. The difference in the adsorption configuration and presence patterns of C atoms on six Co surfaces is derived from the structure sensitivity of different Co surfaces.

### 2.2. Electronic Property of C Atom on HCP Co Surfaces

To gain a deeper understanding of the nature of C deposition on Co surfaces, the electronic properties of C atom adsorbed on hcp Co surfaces were studied. The difference in charge density for the different surfaces was calculated by

(1) $$\Delta \rho ={\rho_{C/}}_{slab}-\rho_{C}-\rho_{slab}$$

where $\rho_{C/\mathrm{slab}}$ represents the charge density of the C adsorbed on Co surface, $\rho_{\mathrm{slab}}$ represents the isolated C atom, and $\rho_{C}$ represents the clean Co surface.

**Figure 2:**
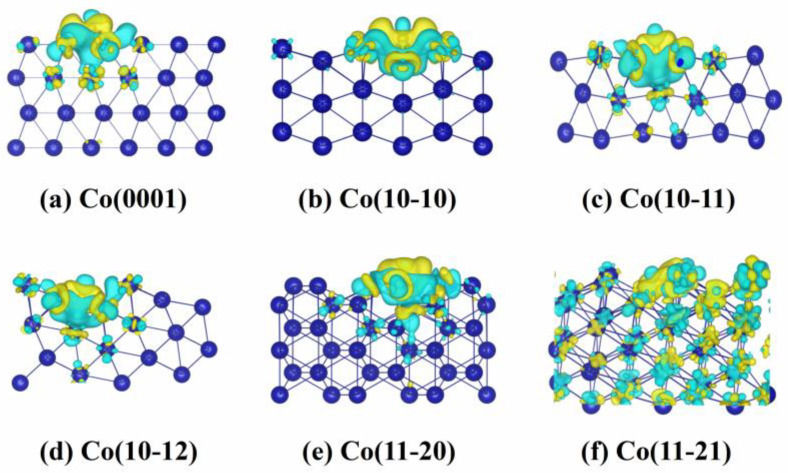
Isosurfaces of charge density difference at 0.02 eÅ^−3^ of C atom at favorable sites on six Co surfaces. The Co atoms are colored blue. Yellow and green regions indicate charge accumulation and depletion, respectively.

The charge density difference of the C atom at favorable sites is shown in Figure 2. An electron-depleted region (green) appears between the C atom and Co surfaces, while there is an electron-accumulated region (yellow) around the C atom, suggesting that electrons are transferred from Co surfaces to the C atom. The amount of charge transfer was quantified by Bader charge analysis [38,39], as shown in Table 1. It is evident that more electrons are transferred from the wrinkled Co(10-10), Co(10-11), Co(10-12), Co(11-20), and Co(11-21) surfaces (0.68 *e*, 0.83 *e*, 0.82 *e*, 0.80 *e*, and 0.82 *e*, respectively) than from the flat Co(0001) surface (0.62 *e*). With the increase in the degree of surface corrugation, the accumulation of electrons between the C atom and Co surfaces increases, corresponding to the lower adsorption energy. For example, the Co(10-11) surface with the lower adsorption energy of −8.29 eV transferred 0.83 e to the C atom.

**Figure 3:**
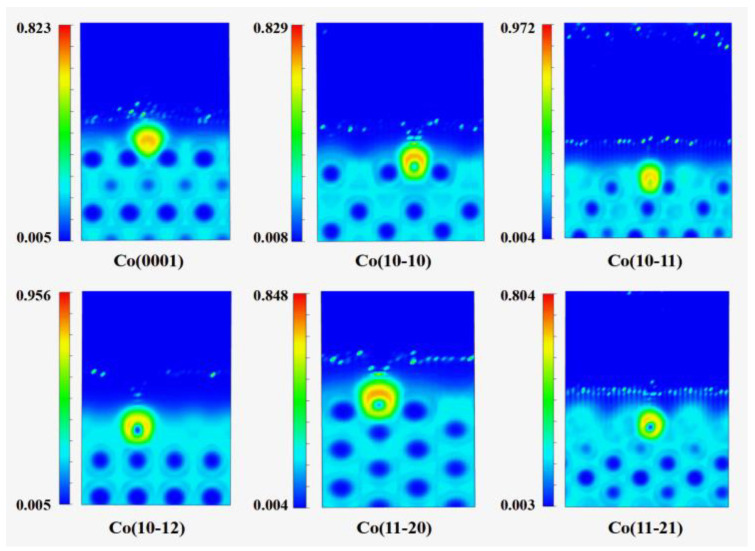
The electron localization function (ELF) for C atom at favorable sites on six Co surfaces.

**Figure 4:**
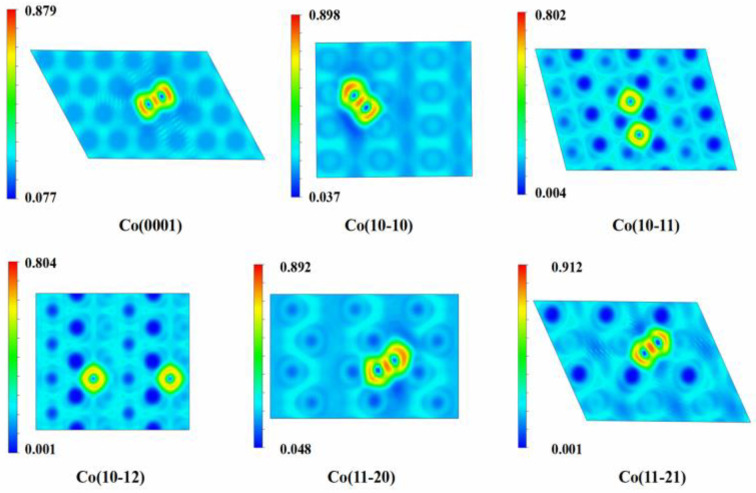
The electron localization function (ELF) for two C atoms at favorable sites on six Co surfaces.

To gain deeper insights into the bonding nature of carbon atoms on various cobalt surfaces, the electron localization function (ELF) analysis for a single C atom and two C atoms adsorbed at their most stable sites on six Co surfaces is displayed in Figure 3 and Figure 4, respectively. The central region of the carbon atom exhibits high charge density, which diminishes in the peripheral areas, which is in agreement with the Bader charge analysis described above. The directional characteristics of the bonding between the carbon and cobalt atoms are pronounced. On the Co(10-11) and Co(10-12) surfaces, the two C atoms adopt the most stable configurations in a dispersed pattern. There are no interactions between these carbon atoms; instead, ionic bonding is observed between each carbon atom and the Co surface. For Co(0001), Co(10-10), Co(11-20), and Co(11-21) surfaces, the electrons are highly localized in the region between the C atoms due to electron sharing, which is an indication of covalent bonding. The bond order, which quantifies the strength of interactions between a single C atom and two C atoms with Co surfaces, follows the order Co(10-11) > Co(10-12) > Co(11-21) > Co(11-20) > Co(10-10) > Co(0001).

**Figure 5:**
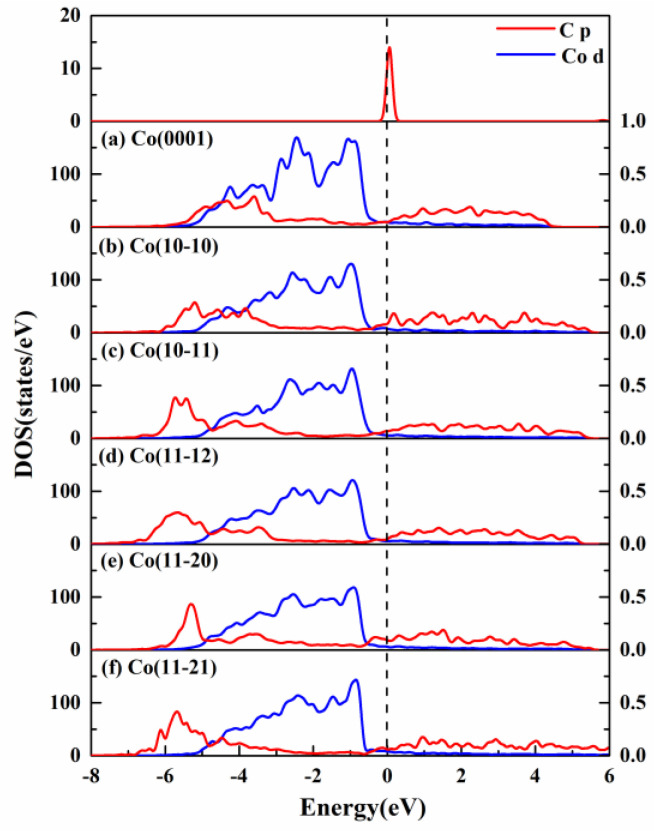
The projected density of states (PDOS) onto *p* orbitals of C atom and d orbitals of Co atom on six hcp Co surfaces. Zero is set at the Fermi energy. The *p* and *d* orbitals are labeled with red and blue lines, respectively.

The projected density of states (PDOS) for the C atom adsorbed on hcp Co surfaces was calculated, as shown in Figure 5. For the isolated C atom, a peak is observed around the Fermi level of the *p* orbital. The *d* orbitals of Co atoms on six Co surfaces are mainly distributed in different peak shapes below the Fermi level, suggesting significant differences in active sites. After C adsorption, the *p* orbitals of the C atom on various Co surfaces become broader and experience a downward shift, resulting in hybridization with the *d* orbitals of the Co atom. The stronger interaction between the C atoms and Co surfaces results in more electrons transferred from Co surfaces to C atoms. As a result, more electrons further induce the *p* orbitals of the C atom to shift toward lower energies, ultimately leading to a lower adsorption energy of the C atom.

For the Co(10-11), Co(11-12), Co(11-20), and Co(11-21) surfaces, the *p* orbitals of the C atoms shift toward the lower energies with peaks in the range of −7 to −5 eV, where the obvious downward movement than that of the Co(0001) and Co(10-10) surfaces reveals the stronger interaction between C atoms and Co surfaces, which is in line with the trend of their adsorption energies.

### 2.3. C Diffusion Behavior on HCP Co Surfaces

**Figure 6:**
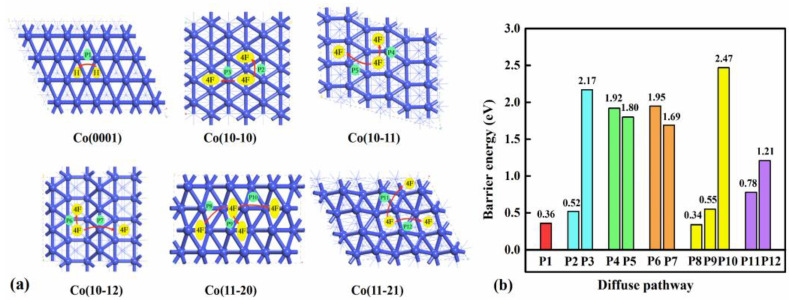
(**a**) Diffusion pathways and (**b**) barriers energy of C atom on hcp Co surfaces.

The diffusion kinetic properties of the C atom on hcp Co surfaces determine the stability of C atom deposition on the Co surfaces and the possibility of C atom aggregation. The most stable adsorption sites for C atoms on different Co surfaces have been determined based on the above calculation. Then, possible diffusion pathways between neighboring favorable adsorption sites were considered, as shown in Figure 6. The transition state structures in the diffusion pathways of the C atom between the most stable sites on six Co surfaces were presented in the Supplementary Materials. For the flat Co(0001) surface, the diffusion path from the stable H site to the nearest H site is labeled path 1 (P1). For the C atom at the stable 4F site on the remaining wrinkled surfaces, there are two possible migration paths: along the surface grooves toward the nearest 4F site following paths of P2, P4, P6, P8, P10, and P11 and across the surface wrinkles toward the adjacent 4F site following paths of P3, P5, P7, P9, and P12.

For the Co(0001), Co(10-10), Co(11-20), and Co(11-21) surfaces, the lower diffusion barriers with 0.36, 0.52, 0.34, 0.55 and 0.78 eV caused by weaker C adsorption of Co surfaces drive C atom to move and aggregate to form C_2_ dimers. Conversely, Co(10-11) and Co(11-12) surfaces exhibit higher diffusion barriers of 1.92, 1.80, 1.95, and 1.69 eV, which are unfavorable for the diffusion of C atoms due to strong interactions between C and Co surfaces. Therefore, as the C coverage increases, the C atoms remain dispersed on Co(10-11) and Co(11-12) surfaces. This conclusion confirms the correctness of the stable adsorption structures of C_2_ dimer on different Co surfaces discussed in Section 2.1.

### 2.4. Morphology Evolution of HCP Co Nanoparticles Induced by Deposited C

Co-based catalysts exhibit a series of crystal facets that significantly influence catalytic activity in processes such as Fischer–Tropsch synthesis. Understanding how the C/Co ratio affects the exposure of these facets is crucial for optimizing catalyst performance. The change in the surface energy of C-deposited hcp Co nanoparticles compared to the pure hcp Co surfaces mainly originates from the contribution of C adsorption. Experimental observations have demonstrated that carbon atoms on Co surfaces can couple to form small carbon oligomers under conditions of hydrogen depletion [40]. Based on the above DFT calculation, C dimers on Co(0001), Co(10-10), Co(11-20), and Co(11-21) surfaces exhibit greater energetic stability compared to single carbon atoms, and the difference between the average adsorption energies of C_2_ dimers and single C atoms on Co(10-11) and Co(10-12) surfaces is minimal (0.04–0.15 eV). Therefore, the stable C_2_ structure unit was used as a criterion for evaluating the change in the surface energy of Co nanoparticles induced by C adsorption.

The Wulff construction is a geometric model used in crystallography to determine the equilibrium shape of a crystal’s surface by calculating the surface energy of different crystal faces. It is based on the principle that the surface will adjust to minimize the surface energy. This construction method can be applied to crystal systems of various dimensions and is of great significance in understanding and controlling the morphology of nanomaterials. In our study, we employed the Wulff construction to predict the morphological changes in the hcp cobalt nanoparticles induced by carbon deposition. By calculating the surface energy of each exposed facet of hcp cobalt and applying the Wulff construction, we can visualize how the crystal’s surface adjusts to minimize its total surface energy, leading to changes in the relative proportions of the exposed facets. The Wulffman program in the VEASTA visualization software was used to display the Wulff construction of the equilibrium shape of the C deposited on the hcp Co nanoparticle.

**Figure 7:**
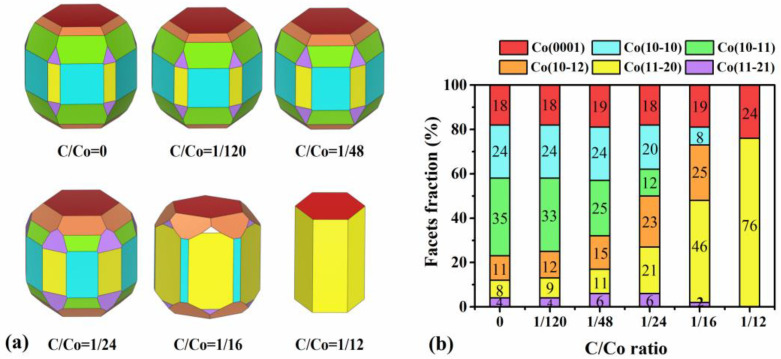
(**a**) Morphologies of C deposited hcp Co nanoparticle and (**b**) facet proportion in different C/Co ratios.

Figure 7 provides a detailed depiction of the ideal morphology of hcp cobalt (Co) nanoparticles, exhibiting a dihedral shape, which is in agreement with previous research [41]. Upon further examination of the surface proportion, it is evident that the Co(0001), Co(10-10), Co(10-11), Co(10-12), Co(11-20), and Co(11-21) surfaces collectively cover the total surface area, with individual contributions of 18%, 24%, 35%, 11%, 8%, and 4%, respectively. As the C/Co ratio varies from 1/120 to 1/16, although the morphology maintains a dihedral shape, the areas of the exposed crystal facets have changed. The Co(0001) maintains its exposure proportion with minimal variation, indicating its stability and lower reactivity towards carbon deposition.

As the C/Co ratio increases, the proportions of Co(10-10) and Co(10-11) exhibit a gradual decline, eventually becoming virtually non-existent at higher carbon concentrations. In contrast, both the Co(10-11) and Co(10-12) surfaces display an opposing trend, with their proportions increasing as the C/Co ratio grows. The Co(11-21) surface, characterized by the minimal exposure proportion, shows negligible variation with the rising C/Co ratio. Upon reaching a C/Co ratio of 1/16, a significant alteration in the surface composition is observed. The proportions of the Co(0001), Co(10-12), and Co(11-20) surface notably increase to 19%, 25%, and 46% of the total surface area, respectively. Conversely, the proportions of Co(10-10), Co(10-11), and Co(11-21) surfaces see a marked reduction in their proportions to 8%, 0%, and 2%, respectively. When the C/Co ratio is further increased to 1/12, the morphology of the hcp Co nanoparticle undergoes a profound transformation, adopting a hexagonal prismatic shape. In this altered morphology, only the Co(0001) and Co(11-20) surfaces remain exposed, with the Co(0001) surface accounting for 24% of the total surface area and the Co(11-20) surface overwhelmingly constituting 76% of the surface area.

This observed evolution in the morphology of the hcp Co nanoparticles can be attributed to the changes in surface energy induced by the increased carbon content. Building upon the work of Chen et al. (2018), who proposed a two-site model for the Fischer-Tropsch (FT) reaction on cobalt catalysts [42], we can infer that the step-edge sites predominantly facilitate CO dissociation and chain growth, while the terrace sites are primarily associated with CH_4_ formation and the hydrogenation of olefins. The distinct active sites on the exposed facets of the catalyst suggest that carbon deposition can be strategically utilized to tailor both the morphology and the active sites of Co-based catalysts. This strategy, in turn, allows for the fine-tuning of the catalyst’s activity and selectivity. Our findings shed light on the potential of carbon deposition as a means to modify the surface properties and morphology of hcp Co nanoparticles, offering valuable insights for the optimization of catalyst performance in FT reactions.

## 3. Conclusions

DFT calculations were employed to uncover the impact of carbon deposition on the adsorption properties and morphological evolution of hcp cobalt nanoparticles. It was found that carbon atoms exhibited significant differences in adsorption energy, electronic characteristics, and diffusion behavior on different crystal facets of hcp Co, leading to changes in the surface energy of specific facets by carbon deposition. Notably, the morphological transformation induced by carbon deposition shows that the Co(10-12) and Co(11-20) facets have an increased proportion at higher carbon contents (C/Co ratio < 1/16), with a corresponding increase in active sites. Our work aims to provide a more comprehensive understanding of the effect of carbon on the morphology of metal crystals, thereby guiding the design of cobalt-based catalysts with excellent performance.

## 4. Models and Methods

### 4.1. Surface Models

For the hcp Co primitive cell, the optimized lattice constants (a = b = 2.500 Å and c = 4.032 Å) are consistent with the experimental data [43] (a = b = 2.507 Å, c = 4.061 Å) and theoretical values [44] (a = b = 2.491 Å, c = 4.023 Å).

The surface models of hcp Co were constructed to mimic the realistic conditions of the catalyst surfaces. These models were used to investigate the adsorption of carbon and its effect on surface morphology. The surface models with the *p* (6 × 4) for Co(0001), *p* (3 × 4) for Co(10-10) and Co(10-11), *p* (2 × 4) for Co(10-12), and *p* (3 × 2) for Co(11-20) and Co(11-21) surfaces were constructed to investigate the carbon deposition, as shown in Figure 1. During the calculations, the top two layers with the adsorbent were fully allowed to relax, while the bottom layer was fixed to mimic the bulk behavior. The vacuum layer was set to 15 Å to avoid the interaction of periodically repeated slabs, ensuring that the calculated properties are not affected by artificial interactions.

The average adsorption energy of the C atom is calculated by the following equation:

(2) $$E_{\mathrm{ads}/av}=\left[E_{\mathrm{nC}/\mathrm{slab}}-E_{slab}-nE_{C}\right]/n$$

where *E*_nC/slab_ is the total energy of the Co surface with *n* adsorbed carbon atoms, *E*_slab_ is the energy of the clean Co surface, and EC is the energy of the C atom in the gas phase.

The surface energy of a clean Co surface is defined by the following equation:

(3) $$\gamma_{\mathrm{slab}}^{0}=\left[E_{\mathrm{slab}}-n_{\mathrm{Co}}E_{bulk}\right]/2A$$

where *E*_slab_ and *E*_bulk_ are the total energies of Co surface and Co bulk, respectively. n_Co_ is the number of Co bulk units in the system, and A is the surface area exposed by the given Co surface.

The surface energy of the Co surface with C atom adsorption was defined as

(4) $$\gamma_{\mathrm{slab}}^{\mathrm{ads}}=\gamma_{\mathrm{slab}}^{0}+n_{C}E_{\mathrm{ads}/\mathrm{av}}/A$$

where $nE_{\mathrm{ads}/\mathrm{av}}/A$ is the change in surface energy caused by C adsorption.

### 4.2. Computational Methods

All the density functional theory (DFT) calculations were performed using the Vienna ab initio simulation package (VASP) [45,46]. DFT is a quantum mechanical modeling method that is widely used in the field of material science to investigate the electronic structure of many-body systems. The exchange correlation of electrons was treated within the generalized gradient approximation (GGA) in the form of the Perdew–Burke–Ernzerhof (PBE) functional [47,48]. GGA provides a balance between computational cost and accuracy, offering a good description of many systems. The projector augmented wave (PAW) method [49,50] was employed for the representation of the wave functions. PAW is a powerful tool for handling electron–core interactions, providing an efficient and accurate way to represent the electronic structure. To ensure that the calculated structures are in their minimum energy configuration, geometry optimizations were carried out with the convergence tolerance energy of 1.0 × 10^−5^ eV and the force convergence criterion of 0.02 eV/Å, respectively. A plane-wave cutoff energy [51] of 450 eV was used to ensure that the wave functions are accurately represented without excessive computational cost. The Brillouin zone was sampled using a special k-points grid of 3 × 3 × 1 mesh with the Monkhorst–Pack method [52], providing a dense enough sampling for accurate integration over the reciprocal space. The climbing image nudged elastic band (CI-NEB) method [53] was employed to search for transition states and analyze the frequency to verify the transition states with only one imaginary frequency.

## Figures and Tables

**Table 1:** The average adsorption energies (*E*_ads/av_, eV), average C-Co bond length (d_C-Co_, Å), and average Bader charge (*q*, e) of the most stable adsorption sites for C adsorption on six Co surfaces.

Facet	*N*_C_	Adsorption Site	*E*_ads/av_ (eV)	*d*_C-Co_ (Å)	*q*
Co(0001)	1	H	−7.04	1.78	0.62
	2,C_2_ dimer	H and F	−7.23	1.98	1.01
Co(10-10)	1	4F	−7.13	1.87	0.68
	2,C_2_ dimer	4F and 3F	−7.35	2.09	1.05
Co(10-11)	1	4F	−8.29	1.91	0.83
	2,C + C	4F and 4F	−8.14	1.90	1.60
Co(10-12)	1	4F	−7.98	1.90	0.82
	2,C + C	4F and 4F	−7.94	1.91	1.61
Co(11-20)	1	4F	−7.26	1.98	0.80
	2,C_2_ dimer	4F and 3F	−7.49	2.04	1.17
Co(11-21)	1	4F	−7.73	1.90	0.82
	2,C_2_ dimer	4F and 3F2	−7.78	2.01	1.07

## Data Availability

Raw data are available from the corresponding author upon request.

## Supplementary Materials

The following supporting information can be downloaded at: https://www.mdpi.com/article/10.3390/molecules29194760/s1. Figure S1: Diffusion pathways and transition state structures of C atoms between the most stable sites on six Co surfaces.
