# Supplementary material for: Adsorption Property and Morphology Evolution of C Deposited on HCP Co Nanoparticles
Source: Molecules. 2024 Oct 8;29(19):4760. doi: 10.3390/molecules29194760 (PMC11478246; doi:10.3390/molecules29194760)
Supplement: Supplementary file 1 [file molecules-29-04760-s001.zip › molecules-3229763-supplementary.pdf]

Supporting Information

Adsorption Property and Morphology Evolution of C deposited on Hcp Co Nanoparticles

Lili Liu [1,*], Yujia shi [1], Jiamin Rong [1], Qiang Wang [2,*], Min Zhong [3]

[1] School of Semiconductor and Physics, North University of China, Taiyuan, Shanxi 030051, P. R. China; lililiu@nuc.edu.cn (L.L.), 2432900889@qq.com (Y.S.), rongjiamin@126.com (J.R);

[2] National Key Laboratory of High Efficiency and Low Carbon Utilization of Coal, Institute of Coal Chemistry, Chinese Academy of Sciences, Taiyuan 030001, P. R. China; wqiang@sxicc.ac.cn (Q.W.);

[3] Chemical Synthesis and Pollution Control Key Laboratory of Sichuan Province, College of Chemistry and Chemical Engineering, China West Normal University, Nanchong, 637002 Sichuan, P. R. China; m_825179076@sina.com (M.Z.)

* Correspondence: lililiu@nuc.edu.cn (L.L.); wqiang@sxicc.ac.cn (Q.W.)

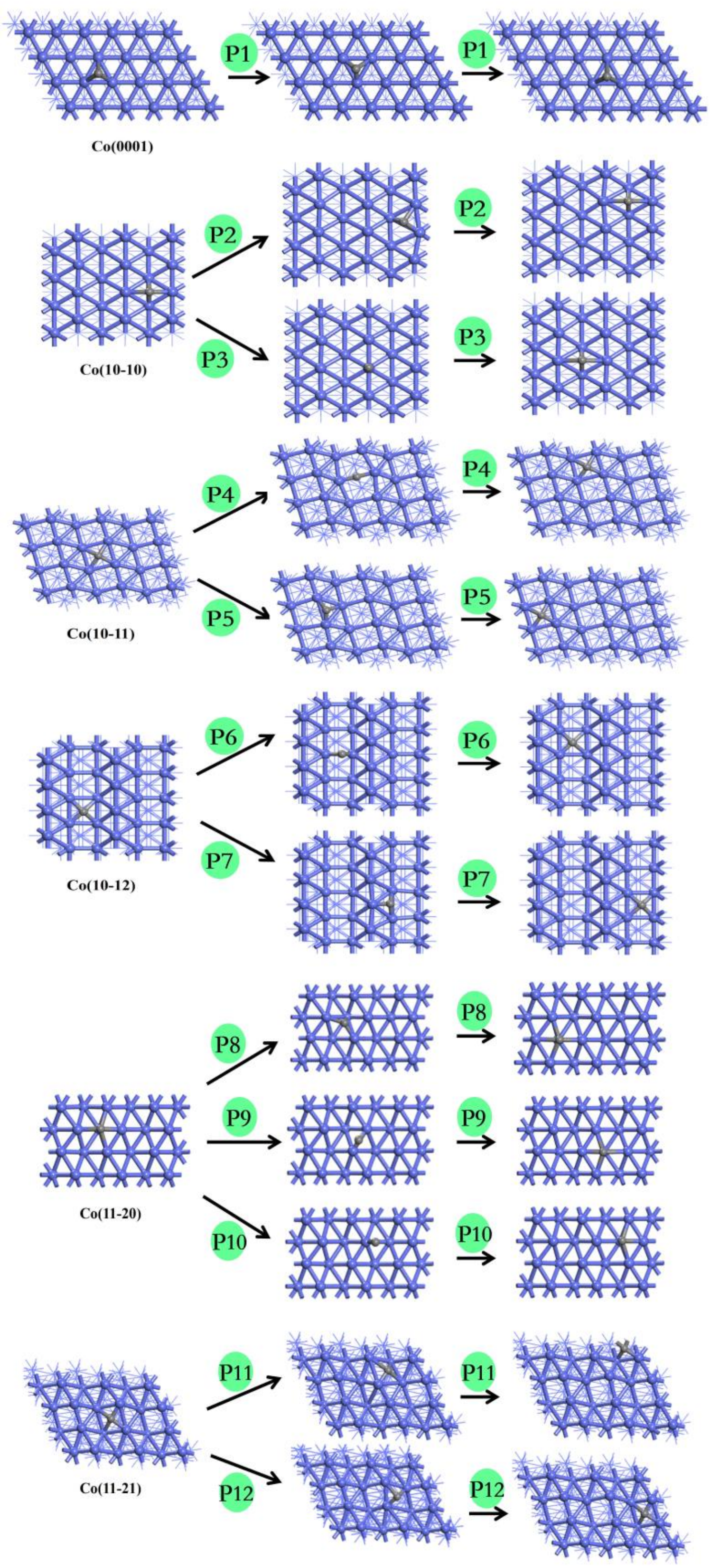

Figure. S1. Diffusion pathways and transition state structures of C atom between the most stable sites on six Co surfaces.
